# Fluidity Investigation of Pure Al and Al-Si Alloys

**DOI:** 10.3390/ma14185372

**Published:** 2021-09-17

**Authors:** Toshio Haga, Shinjiro Imamura, Hiroshi Fuse

**Affiliations:** 1 Department of Mechanical Engineering, Faculty of Engineering, Osaka Institute of Technology, 5-16-1 Omiya, Asahi-Ku, Osaka 535-8585, Japan; 2 Department of Mechanical Engineering, Faculty of Engineering, Graduate School of Osaka Institute of Technology, 5-16-1 Omiya, Asahi-Ku, Osaka 535-8585, Japan; m1m18405@st.oit.ac.jp; 3 Monozukuri Center, Osaka Institute of Technology, 5-16-1 Omiya, Asahi-Ku, Osaka 535-8585, Japan; hiroshi.fuse@oit.ac.jp

**Keywords:** fluidity, die casting, spiral die, peeling of solidification layer, heat shrinkage

## Abstract

Fluidity tests of pure aluminum 1070 and Al-Si alloys with Si contents of up to 25% were conducted using a die cast machine equipped with a spiral die. The effects of the channel gap, die temperature, and injection speed on the fluidity were investigated. When the channel gap was small (0.5 mm), the flow length of the 1070 was minimized, and the fluidity increased monotonically at a gradual rate with increasing Si content. In contrast, larger gaps yielded convex fluidity–Si content curves. Additionally, heating the die had less of an influence on the fluidity of the 1070 than on that of the Al-Si alloy. These results are discussed in the context of the peeling of the solidification layer from the die based on the thicknesses of foils and strips cast by melt spinning and roll casting, respectively. At lower Si contents, heat shrinkage was greater and the latent heat was lower. When the heat shrinkage was greater, the solidification layer began to peel earlier, and the heat transfer between the solidification layer and the die became smaller. As a result, the fluidity of the 1070 was greatest when the channel gap was 0.8 mm.

## 1. Introduction

Aluminum alloys with excellent fluidity are considered suitable for a range of applications, including thin-walled products such as lightweight heat sinks with thin fins. There have been many studies on the factors that affect the fluidity of aluminum alloys, which include the solidification mode [1,2,3,4,5], metal composition [6,7,8,9,10], superheating of the molten metal [4,10,11,12], viscosity [2,12,13,14,15], surface tension [16,17,18], mold materials [1,19,20,21], and mold temperature [22,23,24,25]. The effect of the Si content on the fluidity of Al-Si alloys, including pure Al, has been discussed from the perspective of solidification patterns, such as columnar and equiaxed dendrites. Three types of solidification modes have been presented: (a) pure metals, dilute alloys, and eutectics stopped by pinching because solidification proceeds smoothly from the channel wall; (b) solute-rich alloys with a wide solidification range stopped by choking, in which the flow is choked by the precipitation of equiaxed grains and the accumulation of solid crystallites; and (c) a solidification mode with mixed growth for the other areas [1,2,3,4,5]. The excellent fluidity of pure aluminum initially decreases with the addition of a small amount of alloying elements because of the expanded crystallization range and the change in solidification pattern from planar to mushy. The fluidity decreases with increasing alloying content until a minimum is reached, where the alloy composition exhibits a long crystallization range. The fluidity then increases with further increases in the alloying content until a maximum is reached at the eutectic composition, where the solidification pattern is planar [1,2,3,4,5]. In this way, the fluidity of Al-Si alloys has been discussed with particular attention paid to the solidification mechanism.

In previous research on the fluidity of die-cast alloys, the effect of the peeling of the solidification layer from the die, which occurs as a result of heat shrinkage, on the fluidity has not been considered. When heat shrinkage occurs, the heat transfer between the solidification layer and the die is reduced by the peeling off of the solidification layer from the die. The amount of heat shrinkage depends on the Si content of the Al-Si alloy. It is likely that the change in the heat transfer resulting from the peeling of the solidification layer affects the solidification and consequently the fluidity. If the peeling of the solidification layer occurs early, the heat transfer between the solidification layer and the die is small, and the molten metal solidifies slowly. As a result, the fluidity is large in this case. The peeling of the solidification layer may be affected by the Si content of the Al-Si alloy and casting conditions, i.e., the die temperature, the plunger speed of the die casting machine, and the channel gap of the die. In this way, the peeling of the solidification layer may be the underlying mechanism affecting the fluidity when these factors are varied.

The fluidity of commercial electrically conductive (EC) grade 1070 pure aluminum and Al-Si alloys with different Si contents was tested using a spiral die attached to a die casting machine [26,27,28,29]. The channel gap of the spiral die used to investigate the fluidity was set to 0.5, 0.8, and 1.0 mm to consider the casting of thin products. The fluidity of Al-Si alloys in the thin channel gap was experimentally investigated, but the experimental results are not discussed in detail here. It is known that pure aluminum has excellent fluidity because of its solidification pattern [1,2,3,4,5]. However, the superiority of the fluidity of the pure aluminum became small or was lost under some conditions, an effect that cannot be explained by the solidification pattern. In this paper, the effects of the casting conditions on the fluidity are discussed from the perspective of inducing the peeling of the solidification layer. It is difficult to investigate the peeling off of the solidification layer from the die during die casting. In this paper, the thickness of foils and strips of 1070 and Al-Si alloys cast using the melt spinning method and a roll caster is used as a focal point to discuss the relationship between the peeling and the fluidity.

## 2. Experimental Methods

**Figure 1:**
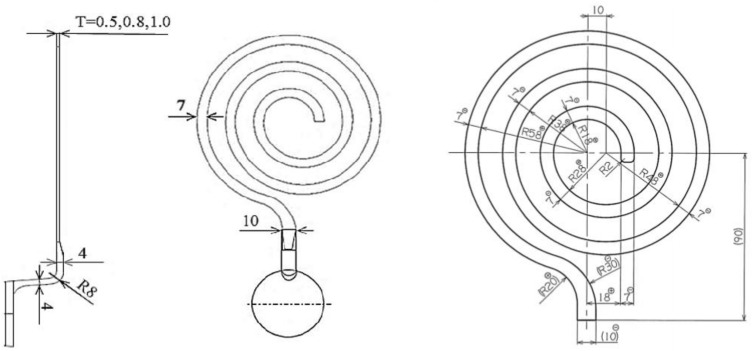
Spiral die used for fluidity test.

A 500 kN cold chamber die cast machine (HC 50F, Hishinuma machinery, Ranzan town, Saitama prefecture, Japan) with an injection power of 100 kN and a sleeve diameter of 45 mm was used in this study [30]. A spiral die was used to investigate the fluidity. The spiral die is shown in Figure 1. The width of the channel of the die was 7 mm, and the channel gap was set to 0.5, 0.8, and 1.0 mm. A crimp was placed at the tip of the spiral test piece for the fluidity test, and beyond this, the channel gap was not filled by the molten metal. The crimp and the unfilled area were not considered as part of the flow path [28]. Only the section with a rectangular cross-section was measured as the flow path length. Plunger speeds of 0.4 and 0.8 m/s and die temperatures of 30 and 150 °C were investigated to observe the effect of these parameters on the fluidity at various Si contents. A mold temperature controller (TT-288, Hishinuma machinery, Ranzan town, Saitama prefecture, Japan) was used to heat the spiral die [31]. Pure aluminum 1070 and Al-Si alloys were used as the target materials. The Si contents of the Al-Si were 1, 2, 4, 5, 6, 8, 10, 13, 15, 17.5, 20, 22.4, and 25%. The pouring temperature was 790 °C. The aluminum alloy was melted in an oxidizing atmosphere using a gas furnace. The melt spinning method [32], a twin-roll caster [33], and a melt drag single-roll caster [34] were used to investigate the relationship between the peeling of the solidification layer and its thickness. The diameter and width of the copper roll used for the melt spinning method and the roll casters were 300 and 100 mm, respectively. The roll speed of the melt spinning method was 55 m/s. The roll speeds of the twin-roll caster and the melt drag single-roll caster were 30 and 5 m/min, respectively.

## 3. Results

### 3.1. Effect of Plunger Speed on Fluidity

**Figure 2:**
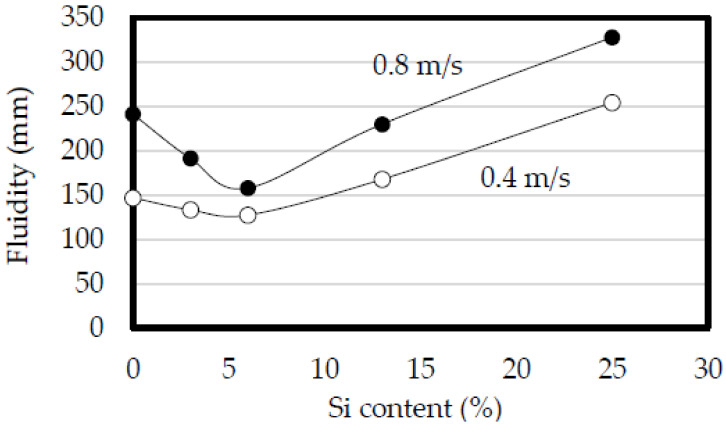
Effect of plunger speed on fluidity. Plunger speed: 0.4 and 0.8 m/s. Die temperature: 30 °C. Channel gap of the spiral die: 0.8 mm.

The effect of the plunger speed on the fluidity is shown in Figure 2 [29]. The pure aluminum 1070 and Al-Si alloys were used. The Si contents of the Al-Si alloys were 3, 6, 13, and 25%. The channel gap of the spiral die was 0.8 mm, and the die temperature was 30 °C. The relationship between the Si content and fluidity was convex, and when the plunger speed was increased from 0.4 to 0.8 m/s, the fluidity of all tested alloys increased, in accordance with prior reported results [35]. Among the considered alloys, the 1070 showed the greatest increase in the fluidity with increased plunger speed, and the Al-6%Si alloy showed the least increase. The increase in the fluidity with the plunger speed was greater at lower Si contents for Si contents below 6%. When the Si content was greater than 6%, the increase in the fluidity with the plunger speed was the same regardless of the Si content.

### 3.2. Effect of Channel Gap on Fluidity

**Figure 3:**
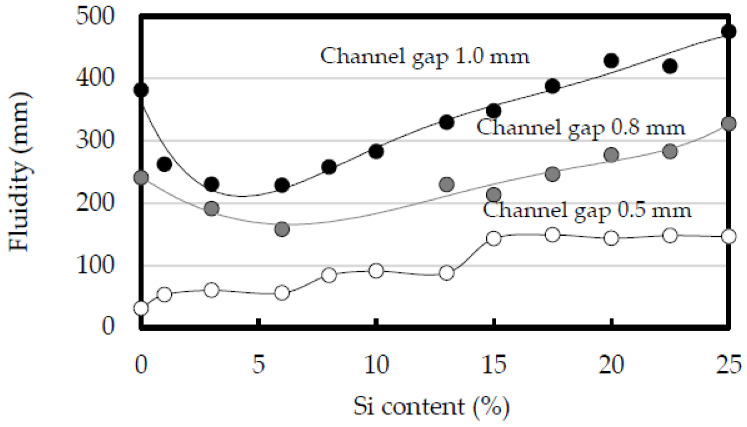
Effect of channel gap on fluidity. Die temperature: 30 °C. Plunger speed: 0.8 m/s. Channel gap: 0.5, 0.8 and 1.0 mm.

The effect of the channel gap of the spiral die on the fluidity is shown in Figure 3 [29]. The die temperature was 30 °C, and the plunger speed was 0.8 m/s. The fluidity of all considered alloys decreased as the channel gap was narrowed. The relationship between the Si content and the fluidity was convex when the die gap was 0.8 and 1.0 mm with a shallower curvature at 0.8 mm [35,36,37]. When the channel gap was 0.8 and 1.0 mm, the fluidity of the 1070 was almost equal to that of the Al-17.5%Si alloy. In contrast, at a channel gap of 0.5 mm, the fluidity gradually increased monotonically with increasing Si content. The fluidity of the aluminum 1070 alloy was the lowest when the channel gap was 0.5 mm. These results demonstrate that the fluidity of pure aluminum is not necessarily greater than that of Al-Si alloys and shows some dependence on the channel gap of the spiral die.

### 3.3. Effect of Die Temperature on Fluidity

**Figure 4:**
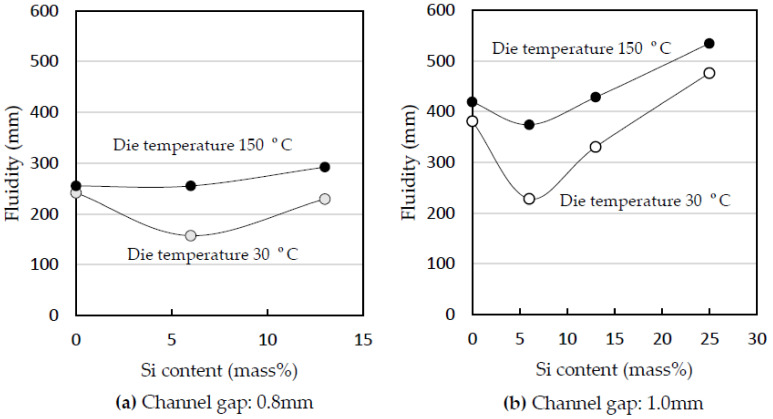
Influence of die temperature on flow length. Plunger speed: 0.8 m/s. Die temperature: 30 and 150 °C. Channel gap: (**a**) 0.8 and (**b**) 1.0 mm.

The effect of the die temperature is shown in Figure 4 [29]. The die temperature was 30 and 150 °C. The plunger speed was 0.8 m/s, and the channel gap was 0.8 mm (Figure 4a) and 1.0 mm (Figure 4b). The fluidity at 150 °C was greater than that at 30 °C [7]. The increase in the fluidity of the 1070 with the die temperature was smaller than that of the Al-Si alloys. The increase in the fluidity of the 1070 at a channel gap of 0.8 mm was smaller than that at 1.0 mm. When the channel gap was 0.8 mm and the die temperature was 150 °C, the fluidity of the 1070 was less than that of the Al-Si alloys, demonstrating that the fluidity of the 1070 is not greater than that of the Al-Si alloys at any die temperature. Among all alloys, the Al-6%Si alloy had the lowest fluidity at a die temperature of 30 °C as well as the greatest increase in fluidity when the die temperature was increased to 150 °C. Increasing the die temperature from 30 to 150 °C had less of an effect on the fluidity of the 1070 than on that of the Al-Si alloys. The effect of increasing the die temperature on the fluidity may decrease with decreasing Si content when the Si content is below 6%. Figure 4 demonstrates that heating the die is not an effective method of increasing the fluidity of the 1070.

## 4. Discussion

### 4.1. Peeling of Solidification Layer

The good fluidity of pure aluminum was previously thought to be a product of its solidification mechanism [3]. It is said that skin formation produces a large fluidity and solidification of the mushy zone produces a small fluidity. However, the results shown in Figure 3 and Figure 4 cannot be explained only by the solidification type. In Figure 3, the superiority of the fluidity of the 1070 was reduced by narrowing the channel gap; at a channel gap of 0.5 mm, the fluidity of the 1070 was less than that of the Al-Si alloys. If the solidification mechanism is the key factor determining the fluidity, the fluidity superiority of the 1070 should not be affected by the channel gap width. It is said that the hindrance caused by crystals with irregular growth surfaces in alloys with a long crystallization range is much greater than that of the comprehensively smooth crystallization interface front of pure metals and eutectic alloys. In solidification of the mushy zone, the presence of free crystals in the liquid can arrest the flow and hence reduce the fluidity [3]. This theory indicates that the fluidity of the 1070 should be superior to that of Al-Si alloys, even Al-6%Si; however, this theory is not in line with the results shown in Figure 4.

**Figure 5:**
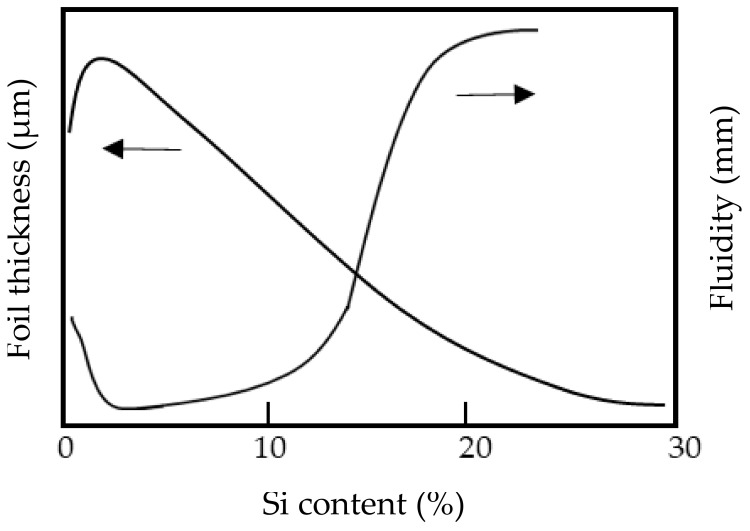
Schematic of foil thickness cast by the melt spinning method and fluidity of gravity casting.

**Figure 6:**
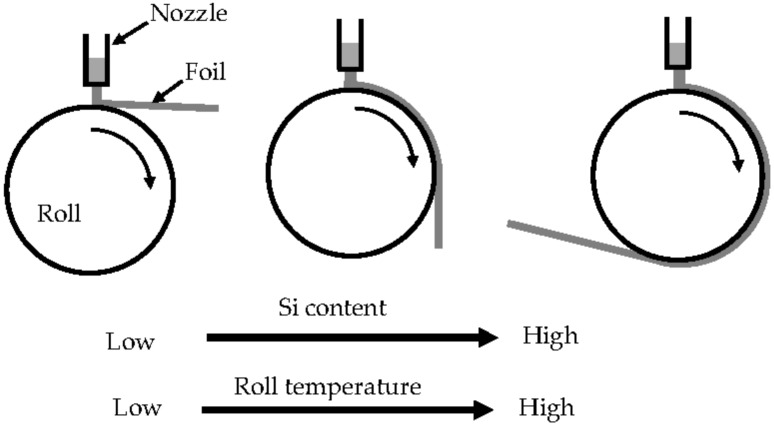
Schematic diagram showing the effect of Si content and roll temperature on the sticking length of foils to the roll.

The relationship between the thickness of Al-Si foils cast using the melt spinning method and the fluidity of the alloy has been previously reported [38]. A schematic diagram of this relationship is shown in Figure 5. As shown in this figure, the foil thickness and the fluidity are inversely related. In this study, we also cast 1070 and Al-Si foils using melt spinning, paying attention to the sticking length of the foil to the roll. The results of this casting are schematically illustrated in Figure 6. The sticking length of the foil increased with increasing Si content, and the sticking length of the 1070 was the lowest. Additionally, the sticking length of the foil increased with increasing roll temperature. These results demonstrate that the Si content and the roll temperature are among the factors determining how the foil sticks to the roll.

**Figure 7:**
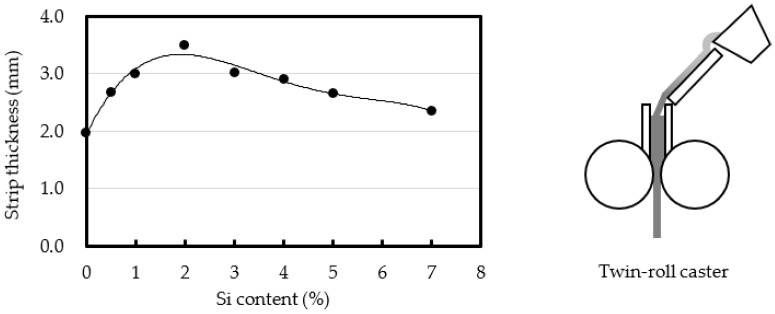
Effect of Si content on the thickness of an Al-Si strip cast using a twin roll caster and a schematic diagram of a twin-roll caster. Roll speed: 60 m/min. Roll load: 0.14 kN/mm.

The effect of the Si content on the strip thickness was also investigated using a twin-roll caster. The results and a schematic diagram of the twin-roll caster are shown in Figure 7 [39]. The relationship between the Si content and the strip thickness showed roughly the same trend as for the foils cast using the melt spinning method. The 1070 strip was the thinnest strip. The roll load of the twin-roll caster was 0.14 kN/mm, which was too small to roll the strip. The strip thickness was almost the same as the sum of the thicknesses of the solidification layers cast by both rolls. It was estimated that the thickness of the solidification layer is also affected by the Si content.

**Figure 8:**
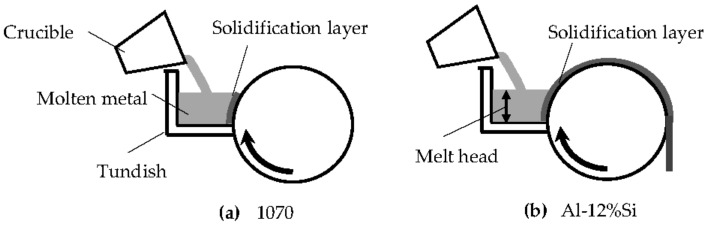
Schematic diagram showing casting conditions for (**a**) aluminum 1070 and (**b**) Al-12%Si strips. Casting speed: 5 m/min.

Figure 8 schematically illustrates the dragging of the 1070 and Al-12%Si solidification layer (strip) using the melt drag method. The position of the tundish was aligned with the center of the roll. The roll speed was 5 m/min, and the melt head was 50 mm. The solidification layer of the 1070 could not be dragged from the molten metal by the roll because the solidification layer of the 1070 peeled from the roll in the molten metal, whereas that of the Al-12%Si was successfully dragged. The solidification shrinkage increased with decreasing Si content in the Al-Si alloys [7]. The results shown in Figure 6 and Figure 8 may be related to the solidification shrinkage. In the melt spinning method, the foil of the 1070 peeled from the roll without sticking to it, and the sticking length increased with increasing Si content. The sticking length may also be affected by the latent heat, which increases with increasing Si content. The solidification layer is cooled more slowly with increasing Si content, causing slow solidification shrinkage. In the melt drag process shown in Figure 8a,b, the 1070 immediately peeled from the roll in the molten metal, whereas the Al-12%Si did not peel in the molten metal and was dragged. The fluidity was considered in the context of the peeling of the solidification layer from the die.

From the results shown in Figure 6 and Figure 8, it can be inferred that the sticking time of the solidification layer decreases with decreasing Si content. The latent heat of the Al-Si alloys increases with increasing Si content [37]. From Figure 5 and Figure 7, when the Si content was greater than 2%, the foil and strip (solidification layer) thicknesses increased and the latent heat decreased with decreasing Si content. However, when the Si content was less than 2%, the foil and strip (solidification layer) became thinner, not thicker, with further reductions in Si content. Thus, there is an inflection point at approximately 2% Si content. Based on conventional knowledge of the effect of the latent heat, the foil and the strip had to become thicker as the latent heat decreased with decreasing Si content, which would mean that the 1070 had to have the largest thickness. As the foil or strip (solidification layer) peels from the roll, the heat transfer between the solidification layer and the roll may become smaller, causing the foil or strip to become thinner. For the 1070 cast by the melt spinning method, it is estimated that peeling started in the molten metal on the roll. When this peeling occurred, the foil and the strip were thinner. This idea was adopted based on the results of the fluidity test.

**Figure 9:**
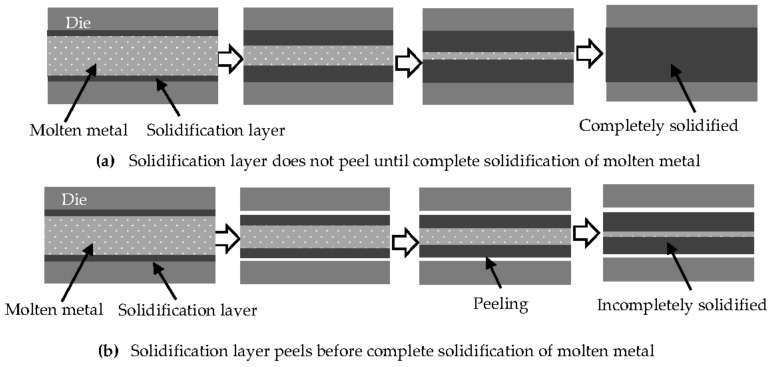
Solidification models where peeling of the solidification layer (**a**) does not occur and (**b**) does occur.

In previous research on the fluidity of aluminum alloys, the peeling of the solidification layer from the die was not considered; this case is illustrated in Figure 9a. In this scenario, peeling does not occur until solidification has finished. However, the present results highlight the possibility that the peeling of the solidification layer from the die occurs before complete solidification, as illustrated in Figure 9b. The remainder of the discussion focuses on the effect of the peeling of the solidification layer and the latent heat on the fluidity.

### 4.2. Plunger Speed

As shown in Figure 2, the increase in the fluidity of the Al-6%Si with increased plunger speed was the lowest among the alloys. On this basis, we propose that the adhesion pattern of the solidification layer of all Al-Si alloy with Si contents of 6% and higher is likely similar to that shown in Figure 9a. In contrast, when the Si content is less than 6%, the solidification layer peeling pattern is likely to be similar to that shown in Figure 9b. In the case shown in Figure 9b, the thickness of the solidification layer increases more slowly with decreasing Si content because the heat shrinkage increases and the peeling of the solidification layer occurs earlier. The gap between the solidification layers becomes thinner with decreasing Si content. As a result, the increase in the fluidity of the 1070 attained by the increasing plunger speed was the largest among the alloys. The thickness of the solidification layer of the Al-6%Si increases more rapidly than for the other alloys, and the gap between the solidification layers thins rapidly in this alloy. Additionally, the Al-6%Si has a smaller latent heat than any of the alloys with Si contents above 6%. As a result, the increase in the fluidity of this alloy with the plunger speed was the smallest.

The superior fluidity of the 1070 over the Al-Si alloy decreases as the plunger speed is reduced from 1 to 0.8 m/s. The fluidity of the 1070 eventually becomes lower than that of the Al-Si alloy, and the fluidity of the alloy increases with increasing Si content for plunger speeds lower than 0.4 m/s. In contrast, the superior fluidity of the 1070 over the Al-Si alloy increases when the plunger speed is higher than 1 m/s. The heat transfer between the solidification layer and the die decreases as the Si content decreases, and the latent heat increases as the Si content increases. The effect of the heat transfer between the solidification layer and the die on the fluidity becomes smaller as the plunger speed becomes slower compared with the latent heat, and the fluidity increases as the Si content increases. The movement speed of the molten metal decreases as the plunger speed decreases. Thus, when the movement speed of the molten metal decreases, the effect of the heat transfer on the fluidity decreases compared to the latent heat.

### 4.3. Channel Gap

**Figure 10:**
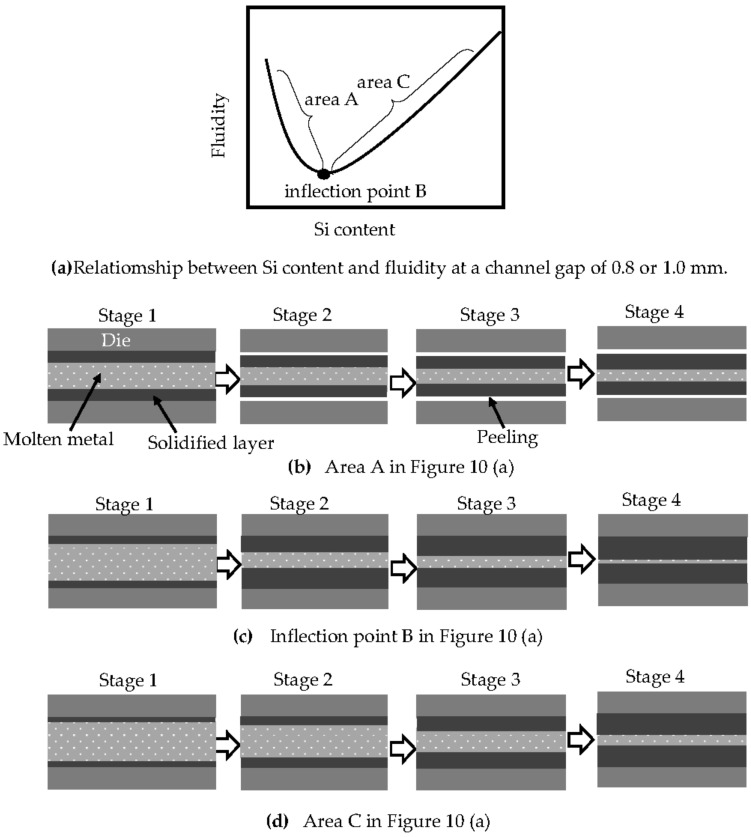
Schematic illustration of the effects of Si content on the solidification of metal in the channel gap of a die. (**a**) Relationship between Si content and fluidity at a channel gap of 0.8 or 1.0 mm. (**b**) Area A in (**a**). (**c**) Inflection point B in (**a**). (**d**) Area C in (**a**).

For channel gaps of 0.8 and 1.0 mm, the relationship between the Si content and the fluidity is as schematically shown in Figure 10a. The solidification patterns at area A, inflection point B, and area C in Figure 10a are illustrated in Figure 10b–d, respectively. In area A (Figure 10b), the heat shrinkage becomes greater as the Si content decreases, and the solidification layer peels off from the surface of the die earlier than at inflection point B and area C. This can be deduced from Figure 6. In area A, the peeling time becomes earlier as the Si content decreases. The thickness of the solidification layer in area A increases more rapidly than at inflection point B or area C until the peeling of the solidification layer from the die occurs, as shown in stage 1 of Figure 10b. The heat transfer between the solidification layer and the die becomes very small after the peeling of the solidification layer occurs. The solidification layer thickens at a remarkably slow rate in comparison with that at inflection point B and area C, as shown in stages 2–4 in Figure 10b. At inflection point B, the latent heat is larger than that in area A, and the thickness of the solidification layer is thinner at stage 1. At stage 2, the solidification layer in area A has peeled from the die, whereas that at inflection point B has not, as shown in Figure 10c. The heat transfer between the solidification layer and the die at inflection point B is greater than that in area A. The solidification layer at inflection point B thickens more rapidly than in area A, in spite of the larger latent heat at inflection point B from stages 2–4 in Figure 10c. The clogging occurs earlier at inflection point B than in area A. In this way, the fluidity in area A is greater than that at inflection point B.

Area C shows the same adhesion pattern as inflection point B, as shown in Figure 10d, and the latent heat increases with increasing Si content. The large latent heat causes the thickness of the solidification layer to grow more slowly, and this growth becomes slower with increasing Si content, causing the gap between the solidification layers to shrink more slowly. As a result, the fluidity increases with increasing Si content in area C. Inflection point B may then represent a change in the contact condition between the solidification layer and the die, from peeling to adhesion. In the area where the Si content is lower than that at the inflection point, the low heat transfer between the solidification layer and the die is the dominant factor determining the fluidity. In contrast, in the area where the Si content is greater than that at the inflection point, the latent heat is the dominant factor. When these two conditions are true, the relationship between the Si content and the fluidity is convex.

**Figure 11:**
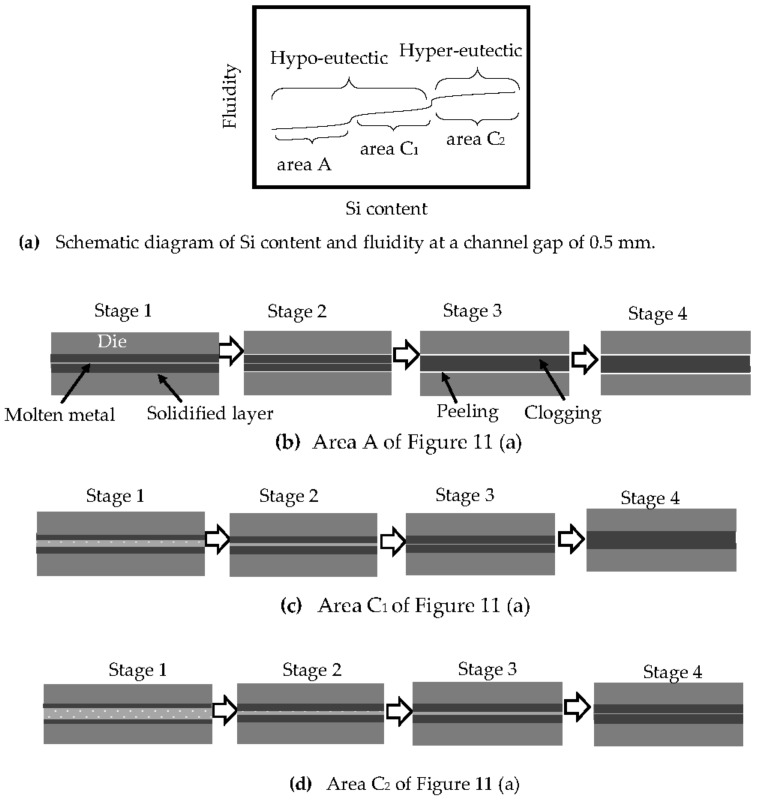
Schematic illustration of the effect of die temperature on the solidification of metal in the channel gap of a die. (**a**) Schematic diagram of Si content and fluidity at a channel gap of 0.5 mm. (**b**) Area A of (**a**). (**c**) Area C_1_ of (**a**). (**d**) Area C_2_ of (**a**).

For a channel gap of 0.5 mm, the relationship between the Si content and fluidity is as shown in Figure 11a. The fluidity decreases with decreasing Si content in the hypoeutectic region, and it is almost uniform in the hypereutectic region. The relationship between the Si content and the fluidity for a channel gap of 0.5 mm was different from that for gaps of 0.8 and 1.0 mm. Unlike the larger channel gaps, at a channel gap of 0.5 mm, the peeling of the solidification layer did not increase the fluidity for Si contents below 6%.

The relationship between Si content and fluidity shown in Figure 3 for a channel gap of 0.5 mm has not previously been reported. This result indicates that the fluidity of pure aluminum is not superior under all casting conditions. It is thought that this result cannot be easily explained by the solidification pattern. When cast thinner than 0.5 mm using a die cast machine, pure aluminum loses its superiority in terms of fluidity. Instead, the hypereutectic Al-Si alloy is suitable for a thin product.

The reason for this is discussed below and shown in Figure 11b–d. In area A in Figure 11a, the thickness of the solidification layer when the peeling occurs increases with decreasing Si content because the latent heat decreases. At this small channel gap width, the gap between the solidification layers, where the molten metal can flow, must be thin because the channel gap is thin, as shown in stage 1 in Figure 11b, and the gap becomes clogged for a short time, as shown in stage 3. In area C_1_ of Figure 11a, the thickness of the solidification layer at stage 1 decreases with increasing Si content as the latent heat exceeds that at the inflection point, and the gap between the solidification layers increases. The gaps at stages 2 and 3 in area C_1_ are wider than those in area A. As a result, the fluidity increases with increasing Si content more in area C_1_ than in area A. As a result, the fluidity is greater in area C_1_ than in area A, even when accounting for the increasing Si content.

In the hypereutectic area, labeled area C_2_ in Figure 11a, the solidification layer may increase more slowly with increasing Si content and latent heat, as shown in Figure 11d. The fluidity of area C_2_ is greater than that of areas A and C_1_. The reduced rate of change in the thickness of the solidification layer with increasing Si content in C_2_, relative to that in areas A and C_1_, may be because the latent heat is sufficiently large. In the hypereutectic region, the number of the primary Si particles increases with increasing Si content, and the viscosity of the semisolid metal increases [37,40]. This may affect the fluidity of hypereutectic Al-Si alloys. Thus, overall, the fluidity of hypereutectic Al-Si remains roughly constant with varying Si content.

### 4.4. Die Temperature

The heat transfer from the solidification layer to the die decreases with increasing die temperature. This means the thickness of the solidification layer increases more slowly and the gap between the solidification layers slowly narrows. As a result, the fluidity increases.

As shown in Figure 4, the 1070 showed the smallest increase in the fluidity when the die temperature was increased, and the Al-6%Si alloy showed the largest. The time that the solidification layer adheres to the die increases with increasing die temperature, as can be inferred from Figure 6. When the die temperature is higher, it takes a longer time for the material to cool to the temperature at which peeling occurs by heat shrinkage.

Additionally, at higher die temperatures, the solidification layer thickens more slowly because the heat transfer from the solidification layer to the die is lower. However, when the Si content is lower than that at the inflection point (Al-6%Si), the adhesion time increases with increasing die temperature, and it again takes a longer time for the material to cool to the temperature at which the peeling of the solidification layer occurs by heat shrinkage. The longer adhesion time has the effect of making the solidification layer thicker. A high die temperature has two effects: one contributes to reducing the thickness of the solidification layer, whereas the other contributes to increasing the thickness.

**Figure 12:**
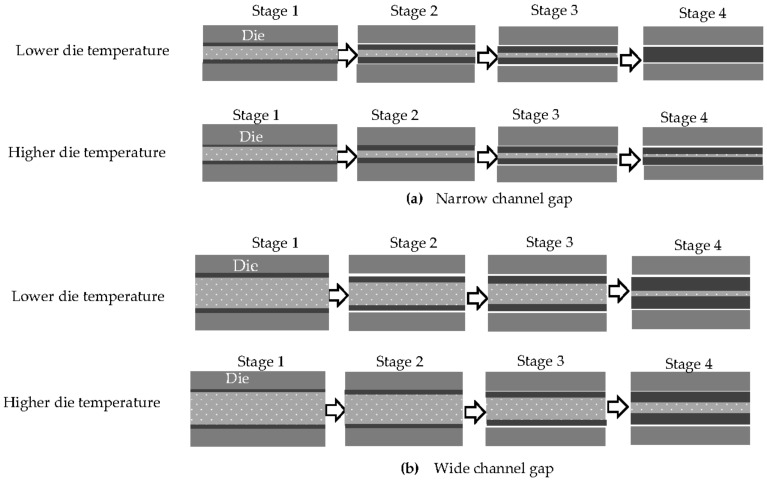
Schematic illustration of the effect of die temperature on the solidification of metal in the channel gap of a die. (**a**) Narrow channel gap. (**b**) Wide channel gap.

When the Si content is less than that at the inflection point (Al-6%Si), the effect of the die temperature on the adhesion time becomes greater as the Si content decreases. The difference between the adhesion times at low and high die temperatures becomes greater with decreasing Si content. The thickness of the solidification layer when the peeling occurs is not much thinner at high die temperatures than at low die temperatures, as the adhesion time increases at higher die temperatures. This means that the increase in the size of the gap between the solidification layers produced by an increase in temperature was smallest for the 1070, as shown in stages 1 and 2 of Figure 12a,b. The rate at which the solidification layer thickens after peeling at the higher die temperature was less than that at the lower die temperature, as the heat transfer from the solidification layer decreases with increasing die temperature. However, the die temperature has less of an effect on the rate at which the solidification layer thickens after the peeling of the solidification layer than it does before. The effect of the die temperature on the fluidity is small after the peeling when the channel gap is narrow because the period from peeling to clogging is short. This is depicted in stages 3 and 4 of Figure 12a,b. When the channel gap is wide, the period from peeling to clogging increases. Therefore, the die temperature has a greater effect on the fluidity when the channel gap is wider, as shown in Figure 12b. In this way, the 1070 showed the smallest increment in fluidity when the die temperature was increased.

When the Si content was greater than that at the inflection point (Al-6%Si), the die temperature had little effect on the adhesion period of the solidification layer. The heat transfer between the solidification layer and the die became smaller, and the solidification layer thickened slowly. Consequently, the gap also narrowed slowly. As the result, the fluidity increased.

The effect of the heat transfer on the increasing speed of the thickening of the solidification layer becomes greater as the latent heat becomes smaller. As a result, at the inflection point, the change in fluidity with temperature was the largest, as shown in Figure 4.

It is common sense when die casting aluminum alloys that increasing the die temperature is very useful. However, in the case of die casting pure aluminum, it became clear that increasing the die temperature is not useful for increasing the fluidity of the pure aluminum.

## 5. Conclusions

The effect of the peeling of the solidification layer from the die on the fluidity of Al-Si alloys was discussed to elucidate the cause of the excellent fluidity of pure aluminum. It has been previously reported that the thickness of foils cast by the melt spinning method is inversely related to the fluidity. The sticking length of the foil was found to increase with increasing Si content, and the sticking length of the 1070 was the lowest among the alloys. Additionally, the sticking length of the foil increased with increasing roll temperature. The relationship between the Si content and the thickness of strips cast by a twin-roll caster showed roughly the same trend as for the foils cast using the melt spinning method, and the 1070 strip was again the thinnest strip. Based on these results, the effect of the peeling of the solidification layer on the fluidity was discussed.

The fluidity of pure aluminum 1070 and Al-Si alloys during die casting was measured using a spiral die. To explain the effect of the channel gap of the die and the die temperature on the fluidity, the peeling of the solidification layer from the die was considered as a potential mechanism driving this effect.

(1) The superiority of the 1070 fluidity to the Al-Si alloy decreased as the plunger speed decreased. This indicates that the effect of the heat transfer on the fluidity decreased compared with the latent heat as the plunger speed decreased.
(2) The relationship between the Si content and fluidity was downward convex when the channel gap was 0.8 or 1.0 mm, whereas it rose monotonically to the right when the gap was 0.5 mm. This shows that pure aluminum does not have superior fluidity to the Al-Si alloys at a very thin channel gap. This result could be explained by the solidification layer peeling from the die earlier at lower Si contents.
(3) The effect of the die temperature on the fluidity was investigated at die temperatures of 30 and 150 °C. The fluidity was greater when the die temperature was 150 °C. The increase in the fluidity of the pure aluminum 1070 with increasing temperature was smaller than that of the Al-Si alloy. It became clear that increasing the die temperature is not useful for increasing the fluidity of pure aluminum. This result could be explained by the solidification layer sticking to the die for longer times at higher die temperatures.
(4) The inflection point in the relationship between Si content and fluidity represents the point at which the dominant factor determining the fluidity changes. In the region of lower Si content to the left the inflection point, the peeling of the solidification layer from the die is the dominant factor, whereas in the region of higher Si content to the right of the inflection point, the latent heat is the dominant factor.

As mentioned above, the effects of casting conditions on the fluidity of die-cast 1070 and Al-Si alloy can be explained by introducing the idea of the peeling of the solidification layer from the die. This peeling affects the heat transfer between the solidification layer and the die. In the wide range of applications of die casting of 1070 and Al-Si alloy, the results of this study may be useful to refine the guidelines for attaining good fluidity, especially for the die casting of thin products.
